# Analysis of gene expression profiles associated with glioma progression

**DOI:** 10.3892/mmr.2015.3583

**Published:** 2015-04-01

**Authors:** GUOZHANG HU, BO WEI, LINA WANG, LE WANG, DALIANG KONG, YING JIN, ZHIGANG SUN

**Affiliations:** 1 Departments of Emergency Medicine, China-Japan Union Hospital of Jilin University, Changchun, Jilin 130033, P.R. China; 2 Departments of Neurosurgery, China-Japan Union Hospital of Jilin University, Changchun, Jilin 130033, P.R. China; 3 Departments of Ophthalmology, China-Japan Union Hospital of Jilin University, Changchun, Jilin 130033, P.R. China; 4 Department of Opthalmology, The First Hospital of Jilin University, Changchun, Jilin 130021, P.R. China; 5 Department of Neurology, Jilin Oilfield General Hospital, Songyuan 131200, P.R. China; 6 Department of Neurosurgery, Affiliated Hospital of Inner Mongolia University for The Nationalities, Inner Mongolia 028007, P.R. China

**Keywords:** glioma progression, gene expression profile, differentially expressed genes, kyoto encyclopedia of genes and genomes enrichment pathway

## Abstract

The present study aimed to investigate changes at the transcript level that are associated with spontaneous astrocytoma progression, and further, to discover novel targets for glioma diagnosis and therapy. GSE4290 microarray data downloaded from Gene Expression Omnibus were used to identify the differentially expressed genes (DGEs) by significant analysis of microarray (SAM). The Short Time Series Expression Miner (STEM) method was then applied to class these DEGs based on their degrees of differentiation in the process of tumor progression. Finally, EnrichNet was used to perform the Kyoto Encyclopedia of Genes and Genomes (KEGG) pathway enrichment analysis based on a protein-protein interaction (PPI) network. A total of 4,506 DEGs were detected, and the number of DEGs was the highest in grade IV cells (2,580 DEGs). These DEGs were classified into nine clusters by the STEM method. In total, 11 KEGG pathways with XD-scores larger than the threshold (0.96) were obtained. The DEGs enriched in pathways 1 (extracellular matrix-receptor interaction), 3 (phagosome) and 6 (type I diabetes mellitus) mainly belonged to cluster 5. Pathway 2 (long-term potentiation), 4 (*Vibrio cholerae* infection) and 5 (epithelial cell signaling in *Helicobacter pylori* infection) was involved with DEGs that belonged to different clusters. Significant changes in gene expression occurred during glioma progression. Pathways 1, 3 and 6 may be important for the deterioration of glioma into glioblastoma, and pathways 2, 4 and 5 may have a role at each stage during glioma progression. The associated DEGs, including *SV2*, *NMDAR* and *mGluRs*, may be suitable as biomarkers or therapeutic targets for gliomas.

## Introduction

Glioma is the most common and most aggressive malignant primary brain tumor in humans, with a morbidity rate of 6/100,000 and a five-year survival rate of 20–30% (1). Gliomas are classified into low-grade types (I or II) with slow or relatively slow growth, and high-grade types (III or IV), with fast growth and spread into normal brain tissue based on the World Health Organization (WHO) grading system (2). Patients with glioblastoma, the highest-grade glioma, survive for no more than one year following diagnosis, as there are still no efficient treatment protocols at present (3). Therefore, early diagnosis of gliomas is crucial for improving the survival of patients.

At present, diagnosis of gliomas is mainly based on histological detection, which, however, cannot reflect the pathological changes at the cellular and molecular levels timely and efficiently. Although the importance of numerous genes, including *TP53*, *PTEN*, *EGFR*, *MDM2*, *CDKN2A* and *CDKN2B* (4–6), during glioma progression has been proven, assays for individual genes/proteins or in combination with histological features are neither predictive of survival of glioma patients nor able to guide therapeutic decisions (7). By constrast, microarrays can provide tremendous information at the transcription level, and are thus likely to reveal dynamic changes in the expression of multiple genes simultaneously. To date, microarray analysis has been successfully used to identify unknown tumor glioma subtypes (8), and gene-based classification has been proven to better correlate with patient survival than histological classification (9).

In the present study, microarray analysis data were subjected to bioinformatics analysis in order to investigate changes at the transcript level that are associated with spontaneous glioma progression, with the objective to discover novel targets for glioma diagnosis and therapy. First, gene expression profile data of glioma samples were compared with non-cancerous samples from epilepsy patients to detect the differentially expressed genes (DEGs) by the significance analysis of microarray (SAM) method. Then, the short time-series expression miner (STEM) method was applied to class these DEGs based on their degree of differentiation in the process of tumor progression. Finally, EnrichNet was used to analyze the enrichment of classified DEGs on Kyoto Encyclopedia of Genes and Genomes (KEGG) pathways based on a protein-protein interaction (PPI) network.

## Materials and methods

### Affymetrix microarray data

The expression profile of GSE4290 (10) was acquired from the Gene Expression Omnibus (GEO, http://www.ncbi.nlm.nih.gov/geo/) database. The platform of those data is GPL570 [HG-U133_Plus_2] Affymetrix Human Genome U133 Plus 2.0 Array. In total, 23 brain tissue samples from epilepsy patients as non-tumor (control) samples and 157 glioma samples, including 26 astrocytoma samples (7 grade II and 19 grade III), 50 oligodendroglioma samples (38 grade II and 12 grade III) and 81 glioblastoma samples (grade IV) were used.

### Data pre-processing

First, the expression microarray data-sets of the glioma and control samples were extracted using the R affy package (http://www.bioconductor.org/packages/release/bioc/html/affy.html). Then, the Robust Multiarray Average (RMA) method, practically supported by the justRMA function of the R affy package, was used to normalize the data through log 2 transformation (11).

### DEG screening and clustering

Significance analysis of micro-array (SAM) of two classes of unpaired measurements was performed for DEG identification using the MeV software (12). Only genes with |log fold change (FC)|≥2 and a false detection rate (FDR) <0.05 were regarded as DEGs between the control and the tumor samples. Regardless of the cell type, DEGs at three grades (II, III and IV) were obtained. Using the Short Time Series Expression Miner (STEM) method specifically designed for the analysis of short time series gene expression data by Ernst and Bar-Joseph (13), these DEGs were classified according to their differential expression degrees during tumor progression.

### Construction of PPI network and KEGG pathway enrichment analysis

The web-accessible online bioinformatics tool EnrichNet (http://www.enrichnet.org/) was used to perform a functional enrichment analysis of the classified DEGs (14) through calculating the overlaps between KEGG pathway and PPI network built with the self-defined gene sets. Unlike the methods (e.g. WebGestalt, http://bioinfo.vanderbilt.edu/webgestalt/ and DAVID, http://david.abcc.ncifcrf.gov/) that only consider overlaps between self-defined gene sets and a known KEGG pathway gene and address the importance of overlaps based on the q-value by statistical tests (eg. one-sided Fisher’s exact test), EnrichNet is an analytical method based on protein-protein interaction (PPI) networks. First, a PPI network was built using the Search Tool for the Retrieval of Interacting Genes (STRING) database (15). Then, by comparing the PPI network with the KEGG database, the similarity degree between PPI and a certain pathway was determined using EnrichNet, which is expressed as the XD-score, with a higher XD-score meaning a greater similarity, indicating higher probability of enrichment in a certain KEGG pathway. To help set the XD-score threshold, EnrichNet also calculated the significance score (q-value) by the classical overlap-based Fisher test, followed by linear regression analysis with XD-score, by which the XD-score corresponding to the q-value of 0.05 was selected as the threshold. Eventually, the obtained Pearson correlation coefficient was 0.85, and the XD-score was set at 0.96.

## Results

### Screening of DEGs

Through microarray analysis, a total of 4,506 DEGs were detected and the number of DEGs increased with the increasing glioma grade, with the largest number of DEGs (2,580) in grade IV cells. The top ten up- and downregulated DEGs at each grade were listed by their FC value (Table I).

### Grade-dependent gene clustering

**Figure 1:**
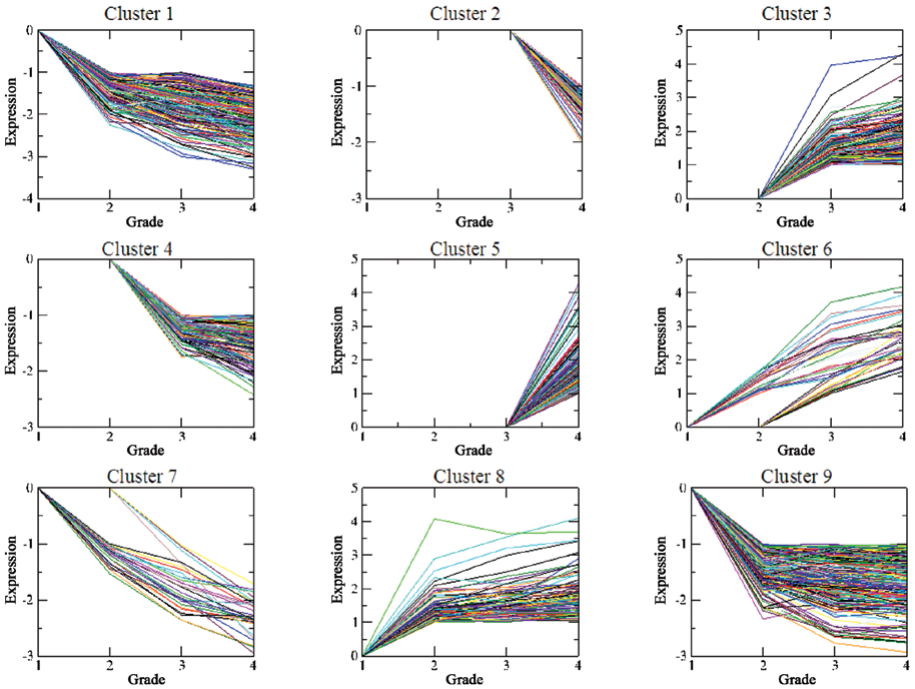
Gene clustering by short time series expression miner analysis. Each line in the figure represents an expression value of the corresponding gene. The abscissa represents the grade of glioma, and the ordinate represents the log_2_ value of the fold change. Negative values indicate downregulated expression, and positive values indicate upregulated expression.

Several genes were not differentially expressed at all the three grades, and the missing expression value was assigned as 0. According to STEM analysis, the DEGs were grouped into nine important gene clusters, which were ranked according to their significance (P-value) (Table II). The differences in the expression values of almost all the DEGs increased with the increasing grade: DEGs in clusters 1, 6, 7, 8 and 9 revealed differential expressions at grade II, while DEGs in clusters 3 and 4 revealed differential expression at grade III, and DEGs in clusters 2 and 5 exhibited differential expression at grade IV (Fig. 1).

### Pathway enrichment analysis of the PPI network

A total of 2,199 genes in the nine clusters were submitted to EnrichNet to construct a PPI network, followed by KEGG pathway enrichment analysis. Finally, 11 KEGG pathways with XD-scores larger than the threshold (0.96) were re-ranked according to their corresponding Fisher q-values (Table III). If q-value <0.05 was set as the threshold, only the first six pathways were enriched.

**Figure 2:**
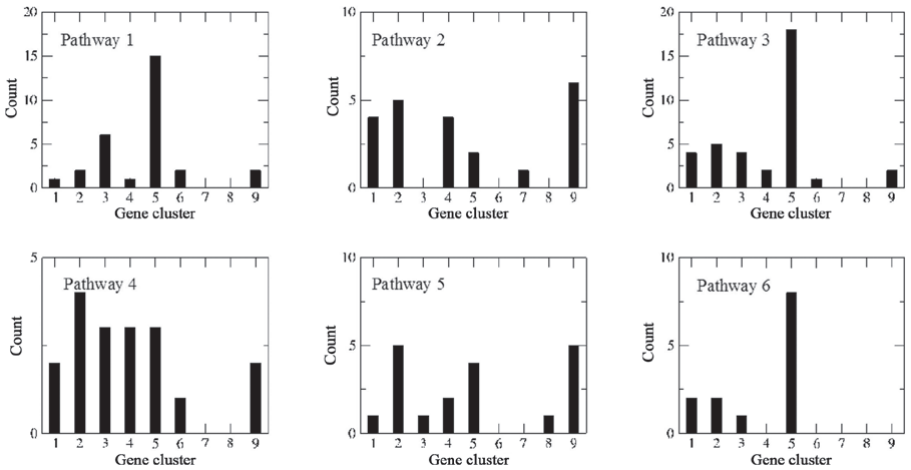
Statistics of overlapping genes between Kyoto Encyclopedia of Genes and Genomes pathways and differentially expressed genes in clusters.

**Figure 3:**
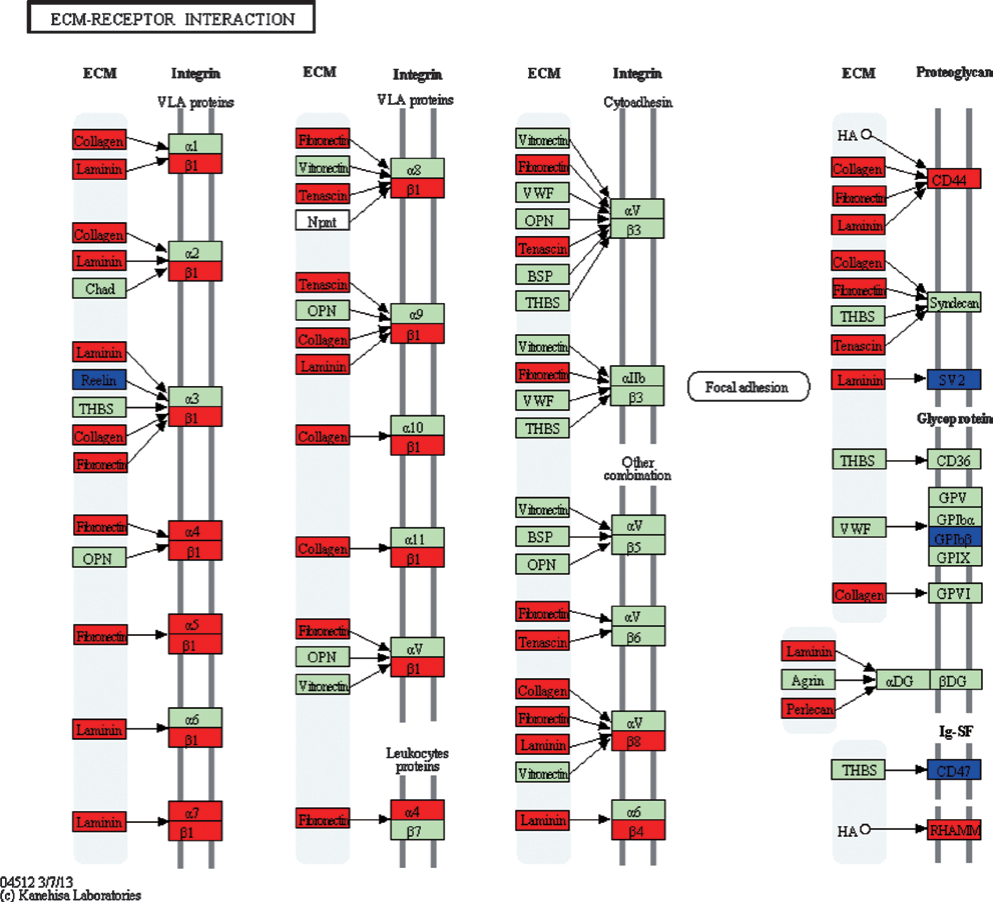
Schematic of the hsa04512: ECM receptor interaction (red indicates upregulated and blue indicates downregulated genes). ECM, extracellular matrix.

**Figure 4:**
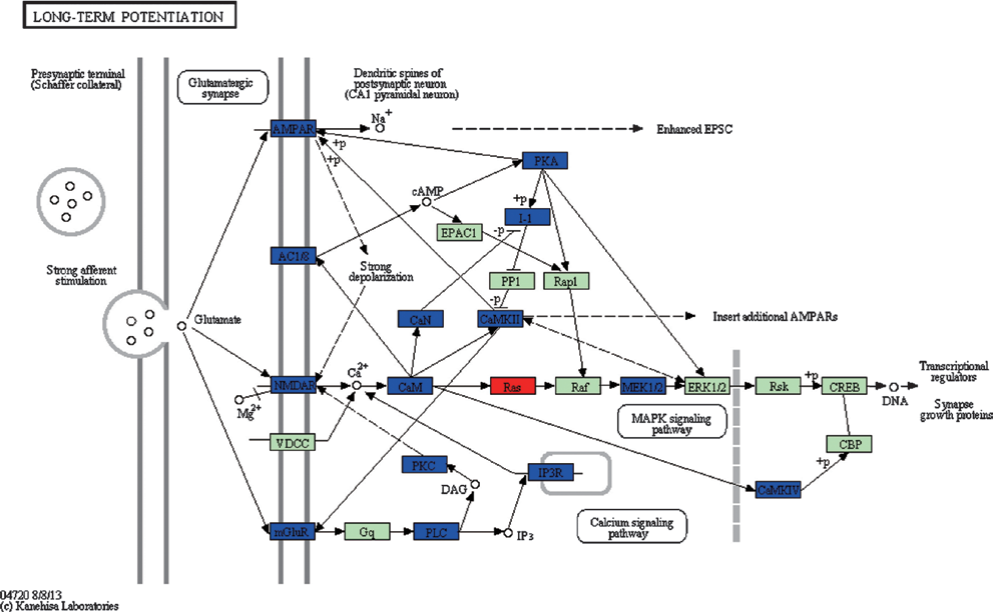
hsa04720: Long-term potentiation (red color indicates upregulated genes and blue color indicates downregulated genes).

For pathway 1 [extracellular matrix (ECM)-receptor interaction] (Fig. 3), 3 (phagosome) and 6 (type I diabetes mellitus), genes participating in these three pathways largely belonged to cluster 5. For example, the collagen-encoding genes (*COL1A2*, *COL3A1*, *COL4A1* and *COL6A2*) and integrin-encoding genes (*ITGA* and *ITGB*) in pathways 1 and 3, and *HLA* genes (*HLA-DPA1*, HLA-DPB1, *HLA-DQB1*, *HLA-DRA*, *HLA-DRB1* and *HLA-G*) in pathways 3 and 6 may have important roles in the development progress of malignant gliomas. For pathways 2 (long-term potentiation) (Fig. 4), 4 (*Vibrio cholerae* infection) and 5 (epithelial cell signaling in *Helicobacter pylori* infection), genes involved in these three pathways belonged to different clusters, indicating that these genes may be associated with the occurrence of glioma. In particular, genes belonging to clusters 1, 8 and 9, which were differentially expressed in grade II glioma cells, accounted for nearly 50% of DEGs in pathway 2 (Fig. 2).

## Discussion

In the present study, the DEGs enriched in pathways 1, 3 and 6 mainly belonged to cluster 5 and were differentially expressed at grade IV, indicating that these pathways and their associated DEGs may have important roles in the deterioration of glioma into glioblastoma. Among these DEGs, integrin-encoding genes (*ITGA* and *ITGB*) were observed to be upregulated in pathways 1 and 3. Taking the ECM-receptor interaction pathway as an example, it refers to the interaction between ECM components and glioma cells, during which cellular matrix receptors function as mediators, and this interaction was previously reported to be responsible for the clinically important features of malignant gliomas, including cerebral invasion and leptomeningeal spread (16). Integrins, a major group of ECM receptors, which are involved in the adhesion and basement membrane invasion of glioma cells (16), were upregulated in this pathway. Collagen-encoding genes, which were markedly upregulated, took a large proportion of DEGs in the first pathway and mostly belonged to cluster 5, suggesting their important role in the deterioration of glioma into glioblastoma. Collagens are primary components in the ECM of most cell types and have been reported to be elevated in gliomas compared to those in normal adult brain cells as well as to have an important role in driving tumor progression (17). In this pathway, a small number of genes, including *SV2* (*SV2B* and *SV2C*), were differentially expressed very early at grade II. Synaptic vesicle glycoproteins (SV2; with the three different isoforms SV2A, SV2B and SV2C) are a group of integral transmembrane proteins (18) with an important role in the development of the central nervous system (19,20). According to a study on patients orally administrated with levetiracetam following surgery, an obvious increase in *SV2* expression was observed in those with a good response (21), suggesting that higher *SV2* levels may indicate a good prognosis. Thus, the significant decrease in *SV2* expression at grade II in this pathway was speculated to correlate with the occurrence of glioma. In addition, human leukocyte antigen (*HLA*) genes, including *HLA-DPA1*, *HLA-DPB1*, *HLA-DQB1*, *HLA-DRA*, *HLA-DRB1* and *HLA-G*, took a large proportion of DEGs in either pathway 3 or 6, particularly in pathway 6, in which these genes were upregulated at grade IV, conforming to the significant expression of *HLA-DQB1* and *HLA-DRB1* in patients with high-grade glioma (HGG) observed by La Torre *et al* (22), and the positive correlation between *HLA-DRB1* and symptomatic cerebral glioma reported by Guerini *et al* (23). Furthermore, introduction of *HLA-G1* or *HLA-G5* into *HLA-G*-negative glioma cells (U87MG) rendered them highly resistant to direct alloreactive lysis, inhibited the alloproliferative response and prevented efficient priming of cytotoxic T cells (24); thus, this gene may contribute to the immune escape in human glioblastoma.

Furthermore, DEGs exclusively detected at grade IV with large FC values (i.e., *IGFBP2* and *GABRA5*) revealed huge variation in expression levels compared to previous grades, which was speculated to be closely correlated with the significant changes during the development of glioma into glioblastoma. *IGFBP2* was reported to be overexpressed in glioblastoma and promote glioma tumor stem cell expansion and survival (25). Gamma-aminobutyric acid (GABA) A receptor, alpha 5 (GABRA5) (26) is a part of the extrasynaptic GABA A-channels and involved in tonic currents (27). The lowest *GABRA5* mRNA levels were found in glioblastoma compared to gliomas of lower malignancy (28), which is consistent with the remarkable decrease in *GABRA5* expression.

Since the DEGs participating in pathways 2, 4 and 5 belong to different clusters, it can be inferred that these pathways and their associated DEGs may have a role at each stage during glioma progression. As the long-term potentiation pathway contained a large proportion of DEGs from clusters 1, 8 and 9, which were differentially expressed in very low-grade glioma cells, it is presumed that this pathway is closely associated with the occurrence of glioma. This pathway has been widely considered as one of the major cellular mechanisms that underlies learning and memory (29). Memory reduction is the most important and distinctive feature in high-grade glioma, thus it can be inferred that memory impairment in high-grade glioma patients is associated with the pathological changes in this molecular process, which may be attributed to the alterations in gene expression in this pathway. Compared to changes observed by Dong *et al* (30) in the LTP enrichment pathway, there were no significant changes observed in the expression of *PP1*, *Rap1*, *Raf* and *ERK1*/*2*, with downregulation of *I-1*, *AMPAR* and *AC1*/*8* expression, and upregulation of *Ras* in the present study. The difference in the expression of these genes may be attributed to the difference in sample size, and should be further investigated. Additionally, Dong *et al* (30) observed the downregulation of *mGluR5* expression in this pathway at grade II, which is consistent with a previous study that reported the impaired learning and reduced CA1 LTP in mice lacking *mGluR5* (31).

In conclusion, long-term potentiation and ECM-receptor interaction were discovered to have an important role in the occurrence and development of gliomas, which may provide a novel and comprehensive view for the treatment of gliomas. The associated DEGs, including *SV2*, *NMDAR* and *mGluRs*, can be considered to be used as biomarkers or therapeutic targets for gliomas. However, the results of the present study require to be further confirmed by additional experiments.

## Figures and Tables

**Table I:** Top ten down- and upregulated differentially expressed genes in grade II, grade III and grade IV glioma samples.

DEGs	Grade II	Grade III	Grade IV
Downregulated	SERPINF1	BRSK1	PRCD
	EPHA5	MAFK	KIAA1324L
	TMEFF2	C6orf114	KCNIP2
	FLJ30594	LOC730125	PPP2R2B
	KIFC2	SEC61A2	TGFA
	FEZF2	BTBD9	ARHGEF4
	STOX2	MEF2C	C10orf84
	AP1S1	ENTPD4	DPY19L2P2
	ADARB1	CACNB3	TMEM16E
	YWHAB	BRUNOL6	PLCL2
Upregulated	TMEM100	SOX11	TOP2A
	LPL	hCG_1815491	IGFBP2
	MTHFD2	TOP2A	PTX3
	SOX11	LPL	hCG_1815491
	BMP2	EGFR	POSTN
	DLL3	PBK	EGFR
	C20orf42	TMEM100	MEOX2
	TIMP4	RRM2	NNMT
	SOX4	TMSL8	RRM2
	EGFR	TIMP4	IBSP

**Table II:** Differentially expressed genes clustered by the short time-series expression miner method.

Cluster	Number of genes	P-value
1	269	1.60×10^−187^
2	374	2.30×10^−54^
3	296	5.20×10^−53^
4	264	8.00×10^−39^
5	478	3.70×10^−36^
6	36	2.80×10^−21^
7	32	1.20×10^−17^
8	127	1.40×10^−7^
9	323	6.30×10^−4^

**Table III:** Enriched Kyoto Encyclopedia of Genes and Genomes pathways for differentially expressed genes in the protein-protein interaction network.

Rank	Pathway	XD-score	Fisher q-value	Overlapping gene number
1	hsa04512: Extracellular matrix-receptor interaction	1.7601	7.58×10^−6^	29
2	hsa04720: Long-term potentiation	1.6560	4.84×10^−4^	22
3	hsa04145: Phagosome	1.0851	4.95×10^−4^	36
4	hsa05110: *Vibrio cholerae* infection	1.9504	6.95×10^−4^	18
5	hsa05120: Epithelial cell signaling in *Helicobacter pylori* infection	1.1594	4.78×10^−3^	19
6	hsa04940: Type I diabetes mellitus	1.3101	1.69×10^−2^	13
7	hsa03030: DNA replication	1.3351	6.49×10^−2^	10
8	hsa04964: Proximal tubule bicarbonate reclamation	1.8351	6.77×10^−2^	7
9	hsa04320: Dorso-ventral axis formation	1.1829	1.17×10^−1^	7
10	hsa04966: Collecting duct acid secretion	1.3551	1.40×10^−1^	7
11	hsa00910: Nitrogen metabolism	0.9779	3.53×10^−1^	5
